# Instant analgesic effect of radial extracorporeal shock wave therapy on primary dysmenorrhoea according to functional magnetic resonance imaging: study protocol for a randomised placebo-controlled trial

**DOI:** 10.1186/s13063-020-4045-5

**Published:** 2020-02-11

**Authors:** Shanshan Liu, Lezheng Wang, Jian Yang

**Affiliations:** 1 Department of Sport Rehabilitation, Shanghai University of Sport, Shanghai, China; 2 Department of Rehabilitation, Shanghai Xuhui Central Hospital / Zhongshan-Xuhui Hospital, Fudan University / Shanghai Clinical Research Center, Chinese Academy of Sciences, 966 Middle Huaihai Road, Shanghai, 200031 China

**Keywords:** Pain, Primary dysmenorrhoea, Radial extracorporeal shock wave therapy wave, Functional magnetic resonance imaging

## Abstract

**Background:**

Primary dysmenorrhoea (PDM) is defined as a series of pain-dominated symptoms during and after menstruation without organic lesions. Nonsteroidal anti-inflammatory drugs and oral contraceptives are usually recommended as first-line therapy for the clinical treatment of PDM, but their widespread long-term application is controversial. Radial extracorporeal shock wave therapy (rESWT) has been widely applied in musculoskeletal rehabilitation because of its secure and noninvasive characteristics and its confirmed effect in improving pain symptoms. This research seeks to explore the efficacy of rESWT for PDM and the changes in brain function of patients with PDM.

**Methods:**

This clinical research will be a randomised, blind, sham-controlled trial. Thirty-six patients with PDM will be randomly divided into the rESWT group (*n* = 18) and the sham rESWT group (*n* = 18). In the rESWT group, treatment will be applied once within 48 h of menstruation at six abdominal myofascial trigger points. The sham rESWT group will receive sham shockwave therapy on the same sites but without energy input. Other dysmenorrhoea-related treatments in both groups will be limited. The main indicators include the short form of the McGill Pain Questionnaire and the Cox Menstrual Symptom Scale. The secondary indicators include the Zung Self-rating Anxiety Scale and Self-rating Depression Scale and functional magnetic resonance imaging (fMRI) changes in brain regions. Results will be evaluated at the screening, at baseline, and before and after treatment, and adverse treatments will be examined. Inter- and intragroup analyses will be performed.

**Discussion:**

This randomised controlled study is designed to explore the immediate efficacy of rESWT for PDM. After rESWT treatment, PDM symptom tests and pain tests, as well as fMRI data, will be investigated for the potential connections between immediate neuroanalgesic mechanisms, which are associated with pain and brain networks. The main results will be used to assess the efficacy of rESWT, and secondary results will focus on improving the neurobiological understanding of disease treatment.

**Trial registration:**

China Clinical Trial Register, ChiCTR1900020678. Registered on 13 January 2019.

## Background

Primary dysmenorrhoea (PDM) involves premenstrual or menstrual pain in the lower abdomen without pelvic organic lesions and may spread to the waist and inner thigh. PDM is also characterised by a series of symptoms such as headache, fatigue and irritability. PDM affects more than 55% of young women worldwide, often with pain so severe that restricts their work and daily activities [1], thus causing considerable personal and socioeconomic burden [2].

Despite the importance of PDM management, clinical treatment is still inadequate. According to the Primary Dysmenorrhea Consensus Guideline, nonsteroidal anti-inflammatory drugs or combined hormonal contraceptives are recommended as first-line treatment for most women with PDM [3]. Nonsteroidal anti-inflammatory drugs hinder the production of peripheral prostaglandins by inhibiting cyclooxygenase, thereby producing analgesic effects [4]. Combined hormonal contraceptives relieve pain by inhibiting ovulation and the growth of endometrial tissue and reducing blood flow and prostaglandins, thus decreasing stress and contractions in the uterus. The analgesic effect of such contraceptives is definite, but long-term use can cause gastrointestinal damage or abnormal hormone secretion. As an alternative treatment, physical factor therapies, such as high-frequency electrical stimulation, ultra-short wave therapy and hot compress [3], have weak practical application because of their obvious side effects, expensive treatment or long treatment cycle and unsatisfactory treatment outcomes.

Radial extracorporeal shock wave therapy (rESWT) involves a kind of mechanical wave that can convert mechanical signals into the target area in vitro into biochemical or molecular signals and induce changes in cell characteristics. A descending pain suppression system that plays a role in analgesia has been accepted all over the world for the extensive treatment of musculoskeletal pain, especially for intractable pain such as chronic periarthritis of the shoulder and stubborn plantar fasciitis [5, 6]. Clinical studies have shown that the acute to chronic transformation of musculoskeletal diseases will lead to a series of peripheral nervous system lesions and even structural changes of the central nervous system, accompanied by different degrees of myofascial trigger point symptoms, such as spontaneous pain and dysmenorrhoea. Moreover, the selection of clinical treatment sites is primarily related to the location of the myofascial trigger point. The clinical study by Huang et al. [7, 8] shows that the pain and related symptoms of PDM can be effectively alleviated in patients with the trigger point of abdominal myofascial pain and who receive local dry and wet acupuncture treatment at the trigger point. Li et al. [9] and Xing [10] confirmed that rESWT can effectively alleviate the symptoms of chronic dysmenorrhoea. In the future, rESWT may be another treatment to complement ibuprofen. However, few studies have been conducted on the immediate analgesic effect of rESWT, and evidence of its central analgesic effect is insufficient.

Tu et al. [11–13] proved that the generation of PDM is related to the abnormal changes in metabolism in brain regions that are related to various aspects of pain management. The major manifestations include increased glucose metabolism in the thalamus, orbitofrontal area and prefrontal area. Conversely, lowered metabolism occurs in the lateral somatosensory motor area. Some imaging studies have confirmed that patients with moderate and severe PDM have structural and functional consistency changes in multiple pain-related brain regions after acupuncture or moxibustion [14–16]. Imaging evidence of functional changes in PDM brain areas is limited by the fact that different interventions have dissimilar effects on brain areas. A recent study [10] showed that rESWT can effectively balance the concentration of blood prostaglandin in women with dysmenorrhoea after the treatment of one menstrual cycle by a trigger point shock wave and can relieve the symptoms of dysmenorrhoea. Furthermore, no adverse events (AEs) occurred during the follow-up after 6 months.

Therefore, we will use rESWT for treatment in the hope of obtaining imaging evidence of analgesia, especially immediate analgesia. In this trial, we will focus on assessing the therapeutic effect of rESWT treatment and sham rESWT treatment. For an in-depth understanding on the mechanism in terms of central analgesia of pain relief by rESWT, the changes in pain state, emotional state and local brain functional areas will be assessed before and after the therapy.

## Methods

### Objectives

Our objectives are to: 1) evaluate the efficacy of rESWT in the treatment of PDM compared with the placebo group; and 2) explore the central mechanism of rESWT in the treatment of PDM according to the results of resting functional magnetic resonance imaging (fMRI).

### Design

**Fig. 1:**
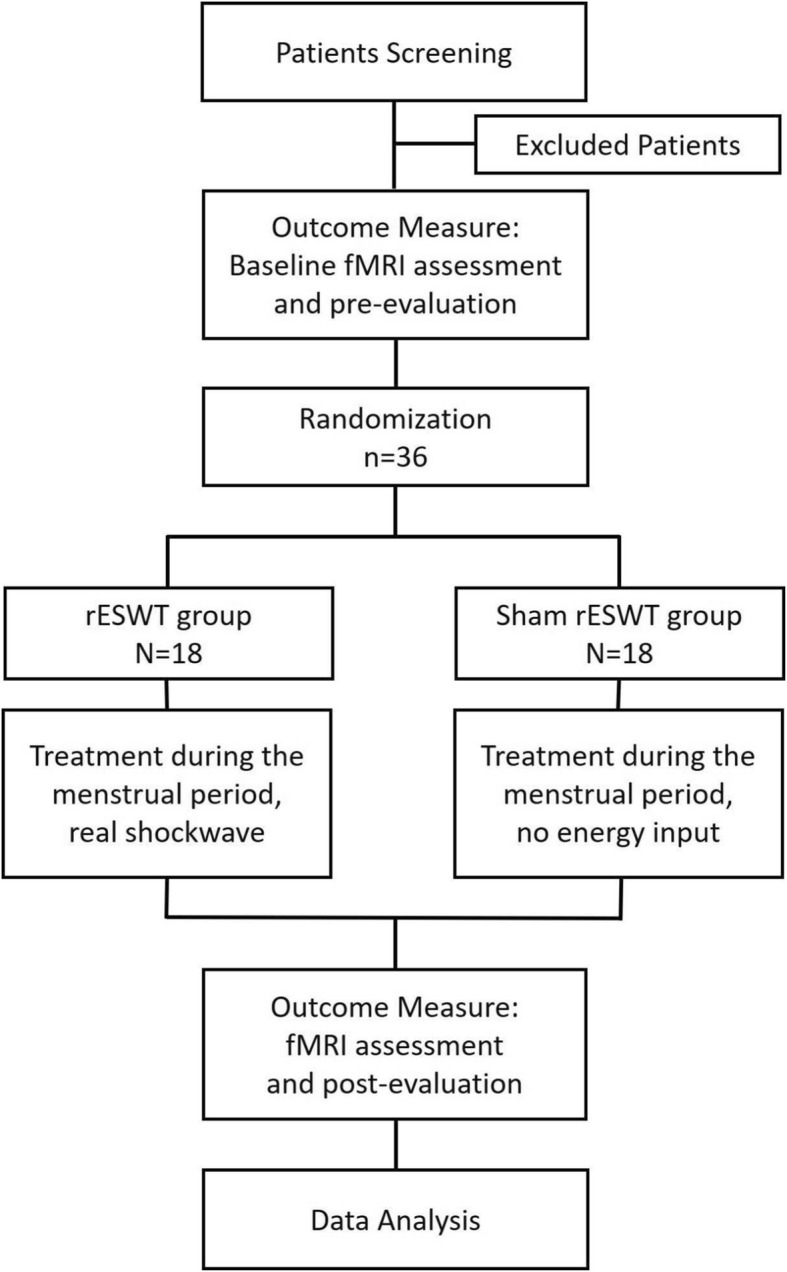
Flow chart of the trial. fMRI functional magnetic resonance imaging, rESWT radial extracorporeal shock wave therapy

This study will be a randomised, assessor- and statistician-blinded, placebo-controlled trial. A total of 36 subjects with PDM who meet the guidelines of the Society of Obstetricians and Gynaecologists of Canada for the diagnosis of PDM will be recruited and randomly assigned to a shock wave group or a sham shock wave group at a 1:1 ratio. Table 1 lists the recruitment process, intervention methods and evaluation, and Fig. 1 depicts the experimental flow chart. The Standard Protocol Items: Recommendations for Interventional Trials (SPIRIT) 2013 checklist is attached as Additional file 1. This study commenced in January 2019 at the Zhongshan-Xuhui Hospital, Fudan University, Shanghai, China.

**Table 1:** Study design schedule

Period	Screening	MRI scan	Treatment	MRI scan	Close-out
Menstrual cycle	0 month	1 month	1 month	1 month	1 month
Inclusion and exclusion criteria	√				
Informed consent	√				
Physical examination	√				
Medical history	√				
Comorbidities	√				
Pelvic MRI	√				
Allocation			√		
SF-MPQ		√		√	
CMSS		√		√	
SAS and SDS		√		√	
Patient compliance					√
Reasons for dropout or withdrawals					√
Adverse events					√
Safety evaluation					√

*CMSS* COX Menstrual Symptom Scale, *MRI* magnetic resonance imaging, *SAS* Zung Self-rating Anxiety Scale, *SDS* Self-rating Depression Scale, *SF-MPQ* Short form of the McGill Pain Questionnaire

### Experimental ethics

The ethics-related aspects of this research were approved by the Institutional Review Board of the Institute of Clinical Trials. The experiment was reviewed and approved by the Ethical Committee of Zhongshan-Xuhui Hospital in January 2019 (approval no. 2018043). The study was registered in the Chinese Clinical Trial Registry in January 2019 (ChiCTR1900020678). This research conforms to the Helsinki Declaration.

Demographics data (height, weight, occupation, family history of dysmenorrhea, years of menstruating, age of onset of dysmenorrhea, menstrual cycle, number of days of menstruation, duration of dysmenorrhea per month (hours), aggravating/alleviating pain factors), questionnaire results, MRI results and signed consent forms will be gathered. All electronic data will be stored in encrypted computers and paper data will be locked in cabinets.

Any modification of the protocol will be documented at www.chictr.org.cn. The research team will disseminate the results of this study by publishing them in peer-reviewed journals.

### Participants

#### Recruitment

Subjects will be recruited from the Shanghai University of Sport, East China Normal University and the Shanghai University of Medicine and Health Sciences through recruitment posters.

#### Inclusion criteria

The inclusion criteria are as follows:

1. Diagnosed as having PDM according to the Primary Dysmenorrhea Guidelines; secondary dysmenorrhea caused by pelvic organ diseases excluded with pelvic MRI [3]
2. 18–30 years old without history of delivery
3. Regular menstrual cycle of 27–32 days
4. Menstrual pain for over 6 months
5. In the past 6 months, the average pain level of menstruation that acts on the central nervous system with conventional treatment was at least four points on a verbal numerical scale (0 = no pain at all, 10 = the most serious pain) [11, 16]
6. No oral contraceptives or drugs that act on the central nervous system within 6 months before treatment
7. Right-handed
8. No previous extracorporeal rESWT therapy
9. Volunteering to participate in the experiment and sign the informed consent form

#### Exclusion criteria

The exclusion criteria are as follows:

1. Mental or neurological disorder
2. History of childbirth
3. Early pregnancy or immediate plans for pregnancy [16]
4. Allergy to the coupling agent
5. Histological changes in the skin or muscle of the treatment site
6. Severe mental illness
7. Serious diseases, such as those of the heart, brain, liver, kidney and haematopoietic system
8. Contraindications to MRI (claustrophobia, pacemaker implants or surgical metal plate in the body)

#### Termination criteria

The criteria for termination of subjects with PDM are as follows:

1. Subjects taking other forms of pain relief, such as extra pain relievers, during the rESWT treatment period of this trial
2. Serious AEs (SAEs)
3. Surgery or hospitalisation for an accident or other diseases
4. Participant request

A reminder will be sent the day before the appointment to ensure participant retention and adherence.

### Randomisation, concealment of allocation and blinding

Participants who meet the inclusion criteria will sign the informed consent form voluntarily and will be eligible for randomisation. They will be assigned randomly to the rESWT group or the sham rESWT group. The random sequence will be generated by a statistician using IBM® SPSS® statistics version 22.0 (IBM Institute, Inc., USA) who is not involved in the intervention or outcome evaluation.

The random number and group assignment will be sent immediately to the independent assessor by another research coordinator via an encrypted electronic file. As much as possible, evaluators will be prevented from communicating with the patients to avoid bias. This process will ensure that randomisation concealment will be competent and will be unaffected by the participants or practitioners.

It is not feasible for the therapist and the patient to be double blind because of the particularity of shock wave therapy. Before signing the informed consent form, patients will be told that they will receive shock wave treatment. Participants will not know their treatment allocation. The therapist will not be blinded, but will not assess the effectiveness of the treatment. Outcome assessors, data managers and statisticians will be blinded to treatment allocation. Prior to the start of the trial, all researchers will be trained to ensure the successful implementation of the blinding procedure.

### Interventions

**Fig. 2:**
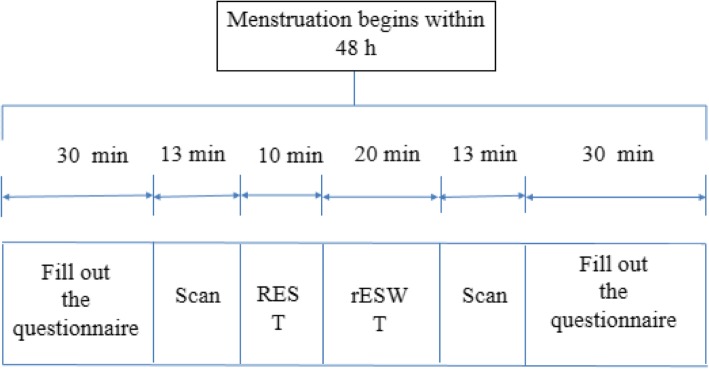
Case study procedure

**Fig. 3:**
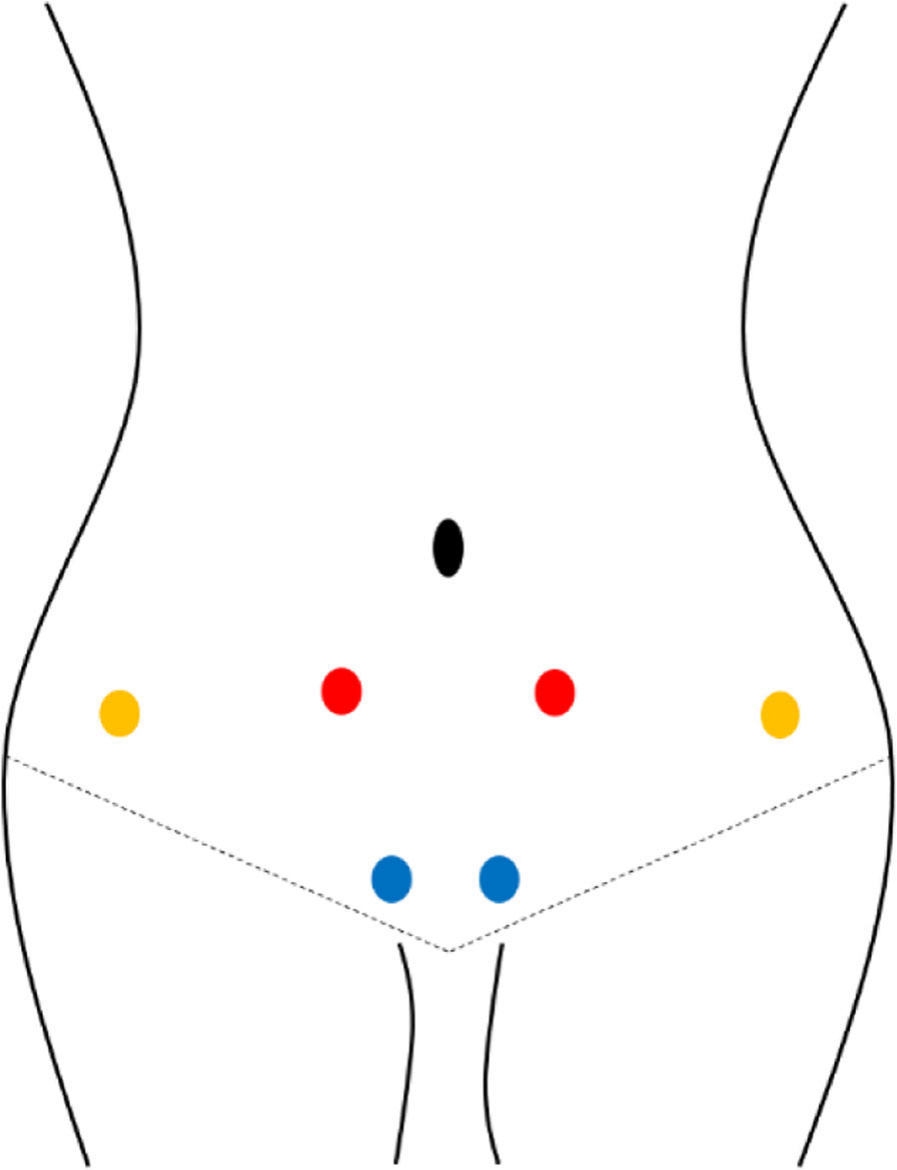
Treatment site of the abdominal muscle fascia trigger point. The blue dots, the rectus abdominis attachment point is located above the pubic symphysis (bilateral); the yellow dots,the intersection of the anterior superior iliac spine with the outer edge of the rectus abdominis (bilateral); the red dots, the anterior superior iliac spine moving inward about 2 cm along a straight line (bilateral)

**Fig. 4:**
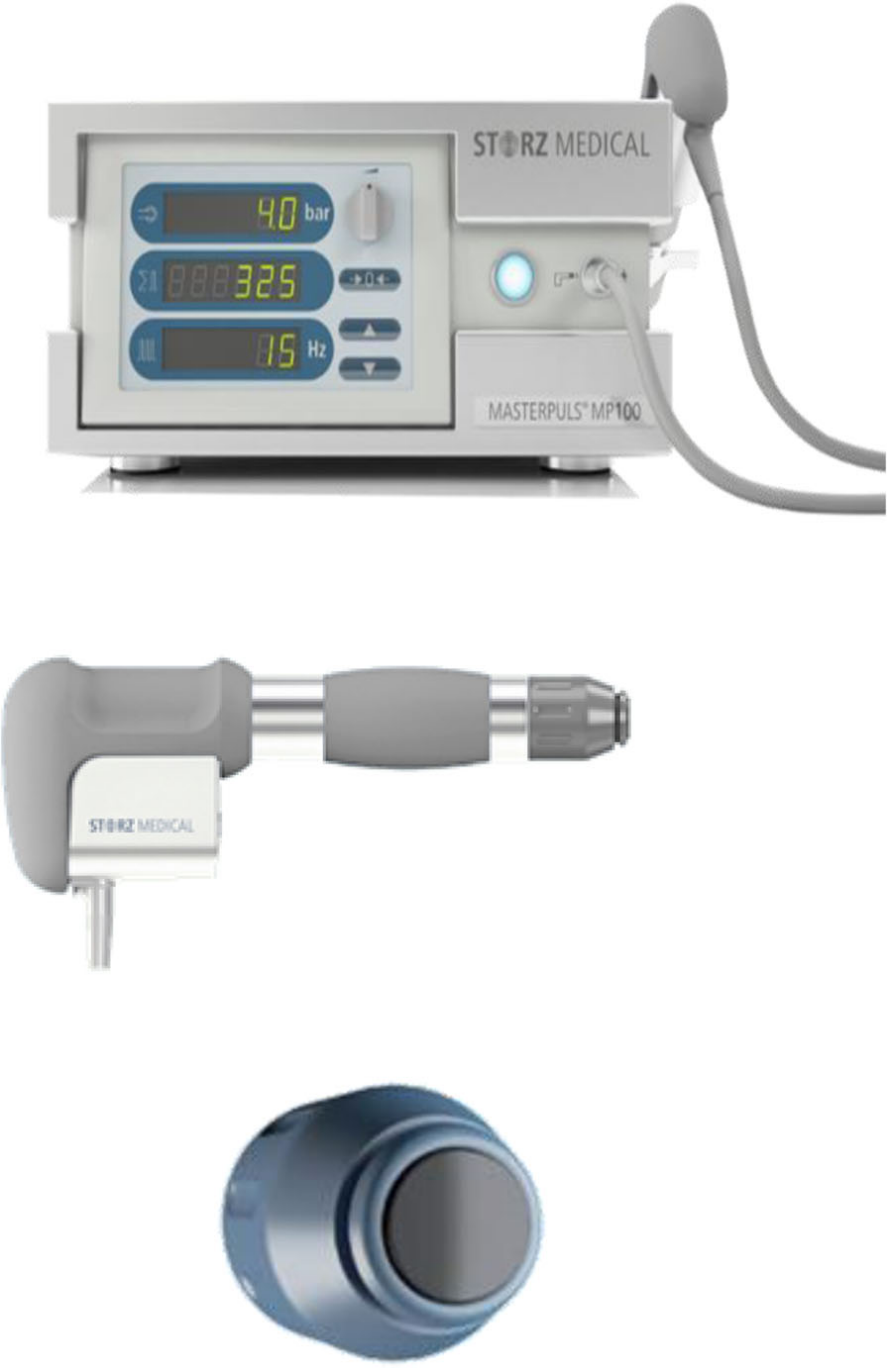
Description of the shockwave instrument. Above is the front view of the shockwave instrument (model: MASTERPULS MP100), and below is the side view of the treatment handle (model: R-SW handpiece)

The time of interventions will be within the first 48 h of the menstrual period (Fig. 2). Trained and qualified practitioners from the Ministry of Health of the People’s Republic of China will perform real and sham rESWT therapy on the participants. Before the intervention, the myofascial trigger points in the abdominal muscles (Fig. 3) will be clinically located by the clinician, and ultrasound coupling gel will be applied on the lower abdominal skin surface (Fig. 2). In the real rESWT group, therapists will use radial shock wave devices based on previous experience to apply 5000 impulses of radial shock waves with a pressure of 1.5 bar at a rate of 10–14 impulses per second for each participant (STORZ MEDICAL AG, MASTERPULS® MP100, C15, diameter 15 mm, Switzerland) (Fig. 4 and Table 2) [9, 10]. Patients in the sham therapy control group will receive placebo intervention with the same sound but no energy input [17]. Moreover, any other treatment to improve menstrual pain will be restricted in both the real and sham rESWT groups during the trial.

**Table 2:** Details of rESWT intervention

Item	Detail	Description
rESWT rationale	Type of shock wave	Pneumatic ballistic extracorporeal shock wave therapy
	Reasoning for treatment	The best treatment option was selected according to our previous study results and other clinical trials of rESWT for primary dysmenorrhoea
	Extent to which treatment varies	None
Details of rESWT	Number of treatment sites per subject	6
	Names of points used	Attachment point of rectus abdominis above the symphysis pubis (bilateral) (blue dot in Fig. 3)
		Intersection of the anterior superior iliac spine with the outer margin of the rectus abdominis (bilateral) (yellow dot in Fig. 3)
		The anterior superior iliac spine shifts inward by approximately 2 cm along the line (bilateral) (location of the red dot in Fig. 3)
	Shock wave intensity	1.5–2.0 bar
	Shock wave frequency	10–14 Hz
	Number of hits from each site	600 times
	Response sought	None
Treatment regimen	Number of treatment sessions	1 time
	Treatment time	Within 48 h of menstruating
Other components of treatment	Details of other interventions administered to the rESWT group	None
	Setting and context of the treatment	Hospital rehabilitation section, outpatient department
Background of practitioner	Profile of the therapist	Specialist in rehabilitation medicine or a resident of more than 1 year under the guidance of the specialist in rehabilitation medicine
Control or comparator	Rationale for the control or comparator in the context of the research question	As a placebo control, a non-energetic sham rESWT was used

### MRI scanning procedure

The Siemens 3.0 T Magnetom Verio MRI system (Siemens Medical, Erlangen, Germany) with 32-channel head coil will be used to obtain MRI data on pain relief after rESWT treatment at the Department of Radiology, Zhongshan-Xuhui Hospital, Fudan University, Shanghai, China. To avoid head movement, foam pads will be used to fix the participants’ heads. The fMRI data will be acquired with a single-shot gradient recalled echo planar imaging sequence with the following parameters: repetition time (TR)/echo time (TE) = 2530 ms/2.98 ms, flip angle = 7, field of view (FOV) = 256 mm × 256 mm and slice thickness = 1 mm. To obtain the resting fMRI data, a whole-brain blood oxygenation level-dependent (BOLD) pulse sequence (TR/TE = 2000/30 ms, flip angle = 90, FOV = 192 mm × 192 mm, voxel size = 3.0 × 3.0 × 5.0 mm, slice thickness = 1 mm and 28 slices) will be used.

### Outcome measurements

#### Primary outcomes

##### SF-MPQ

The primary outcome will be the change in the short form of the McGill Pain Questionnaire (SF-MPQ) score (Table 1). The SF-MPQ is a scale composed of a pain rating index, a visual analogue scale and existing pain intensity to measure pain intensity. The pain rating index consists of 11 sensory and 4 emotional pain descriptors, all of which are rated from 0 to 3 points to indicate the different degrees of no, mild, medium, or severe pain, respectively. This index is a sensitive and reliable pain evaluation method widely used in the assessment of dysmenorrhoea [18].

##### CMSS

The menstrual pain symptoms of participants will be integrally evaluated by the Cox Menstrual Symptom Scale (CMSS), which consists of 18 items or symptoms, including cramps, nausea, vomiting, loss of appetite, headaches, backaches, leg aches, dizziness, weakness, diarrhoea, facial blemishes, abdominal pain, flushing, sleeplessness, general aching, depression, irritability, and nervousness [19]. The scale is mainly used to evaluate the severity and duration of menstrual pain symptoms. All items use a five-level scoring method ranging from 0 to 4 points. The choices for pain severity include painless, mild pain, moderate pain, severe pain, and very serious pain. Patients can evaluate the duration of their pain as 0 h, less than 3 h, 3–7 h, 7–24 h and more than 24 h. The CMSS will be used before and after rESWT treatment.

#### Secondary outcomes

##### Zung SAS

The Self-rating Anxiety Scale (SAS) will be used to measure anxiety-related symptoms in PDM since dysmenorrhoea is often accompanied by emotional change. It is a self-administered questionnaire, with each response using a four-point scale from 0 (none of the time) to 4 (most of the time). A total score above 40 indicates a clinically relevant anxiety disorder [20]. The SAS will be utilised before and after rESWT treatment (Table 1).

##### SDS

The Self-rating Depression Scale (SDS) is a scale used to evaluate changes in terms of depression [20]. In this trial, participants will fill out 20 items based on their actual experience over the past week. The total score for this scale ranges from 20 to 80, with a score above 40 indicating depressive symptoms. This scale will be employed before and after rESWT treatment (Table 1).

### Sample size calculation

The smallest significant differences in clinical treatment are considered to assess the effects of different treatments. SAS 9.4 software was used to design the two groups with better efficacy. The optimal value was defined as 15%. According to previous research results, the effectiveness proportion of shock wave treatment for primary dysmenorrhea is known to be 0.8 [9, 10]. The control group for this experiment was the sham shock wave treatment group. Through previous practice observation, we determined the effectiveness of the control group proportion to be 0.45. The estimated sample size was 30 individuals (15 in each group) to meet the 80% statistical value [21] and the 5% significance level. Considering a 20% drop-out rate, the sample size was finally determined to be 36 participants (18 per group).

### MRI data analysis

An fMRI data processing assistant based on statistical parameter mapping will be used to preprocess the original MRI data (DPARSF, http://rfmri.org/DPARSF) using MATLAB R2015b. After complete preprocessing, data processing and analysis of brain imaging of the amplitude of low-frequency fluctuation (ALFF) will be processed by the resting-state fMRI data analysis toolkit [22]. Afterwards, a comparison, correction and acquisition of the image will be executed via the Rest Slice Viewer software, and the final result will be presented in combination with the specific anatomical position of the corresponding area of MNI coordinates.

The results of fMRI will be interpreted by two different radiologists, and then the rehabilitation doctors will further analyse this in combination with clinical symptoms. Finally, the two doctors will reach a final interpretation through consultation.

### Statistical analysis

Clinical and MRI outcomes will be obtained in this study. Among these outcomes, results from participants for both MRI scans and two clinical evaluations will be included in the results analysis; otherwise they will be considered data from drop-outs. Note that all participants in the experiment will receive our instructions before participating.

Statistical analysis of the clinical outcomes will be accomplished using the SPSS 22.0 statistical software. All tests will be bilateral, and *p* < 0.05 will be considered statistically significant.

Demographic characteristics and baseline information for all participants will be analysed statistically. For continuous data, independent sample *t* tests or Wilcoxon rank sum tests will be applied for analysis with data represented as mean ± standard deviation. If dichotomous data occur, we will use the chi-square test or the Friedman test and represent the data as percentiles.

For post-test results, the paired *t* test or Wilcoxon signed rank test will be employed for continuous data within the group. Conversely, the chi-square test or Fisher’s exact test will be applied for two-part data to compare the differences before and after treatment. For different intervention methods, independent sample *t* tests will be conducted to identify whether the intervention methods show differences for the relief of PDM.

The statistical comparison of MRI data will be conducted in different ways. With the use of the 3.0 T Siemens Magnetom Verio MRI system, each subject will produce two resting state scans. The data processing assistant for resting state fMRI (DPARSF 4.4) software package will be used to process MRI data on the MATLAB 2015b platform and obtain the ALFF values. The mean value of the amplitude of all frequency points within 0.01–0.08 Hz will be calculated; that is, the intensity of the BOLD signal changes will be calculated to obtain the brain map of the statistical parameter of amplitude, which will be used to describe the spontaneous activity of the voxel [23]. SPM12 software will be employed to perform two-sample *t* tests on the two groups of statistical brain maps. The final results will be superimposed on standard avg152 T1 images for display. Finally, the statistical difference of low-frequency amplitudes in the resting state between the two groups will be obtained, and *p* < 0.05 will be considered to indicate a significant statistical difference.

### Security

AEs will be recorded during each operation. The following conditions are defined as SAEs: 1) aggravated illness requiring hospitalisation; 2) disability; and 3) other major medical events. In the event of an SAE, the lead investigator will report to the sponsor within 24 days, and the investigator will intervene as a third party to evaluate whether the AEs are connected to the intervention. If necessary, the appropriate medical services should be provided when the victim asks for compensation. Participants will be withdrawn if the AEs are too severe for them to continue with the trial. However, participants need not withdraw if the AE is unrelated to the intervention or is not a SAE, with respect to the individual wishes of the participants.

Given the safety of rESWT, no safe end point will be predefined because this is considered impossible. However, all AEs will be documented as evidence for safety assessment. Meanwhile, a meaningful number of adverse reactions will be observed.

### Management of study data

In this trial, data managers will use the double-entry method to input data into the Microsoft Excel 2016 software and establish an electronic database. They will also use password protection. The Clinical Research Center of Xuhui District Central Hospital in Shanghai will conduct regular monitoring tests to ensure the integrity and authenticity of all data. Interim data analysis should not be undertaken for any reason at any time. To ensure the confidentiality of the participants, all patients will be made pseudonymous using study identification numbers. Moreover, all researchers will have access to the final database.

### Quality control and monitoring

This study aims to explore the efficacy and mechanism of rESWT for PDM. The Data Monitoring Board is considered a non-essential safety consideration at this time, and similar studies often apply the same approach. Owing to the heavy variety of data tasks, inspectors must monitor the data. According to the actual situation, they will conduct evaluation of the source documents, including medical document charts, related reports and AE records, as well as additional documents, such as agreements, informed consent forms and pathological report forms, all of which will also be under unified management.

## Discussion

Current first-line guidelines for treating PDM advise the use of nonsteroidal anti-inflammatory drugs or oral contraceptives. However, long-term side effects limit the applicability of such treatments [3]. Complementary and alternative therapies include exercise, acupuncture and percutaneous electrical nerve stimulation. In recent years, acupuncture has been used to treat dysmenorrhoea, and studies have found that it can improve symptoms in women with dysmenorrhea and that it is more effective than traditional Chinese medicine or placebo. Other researchers found no advantage for acupuncture when compared with a placebo [3, 24]. A retrospective analysis confirmed the efficacy of high-frequency percutaneous nerve electrical stimulation for dysmenorrhoea. However, high-frequency electrical stimulation may cause local muscle tension and generate adverse reactions, such as headache, nausea, skin flushing or a burning sensation [3]. Such stimulation is limited in its practical application owing to its obvious side effects, expensive treatment or long treatment cycle, unsatisfactory treatment outcome and for other reasons. Therefore, safer and more effective non-drug analgesic schemes must be explored to control PDM.

As far as we know, this work will be the first randomised clinical trial to evaluate the effects of rESWT on pain relief, mental health and changes in brain function in patients with PDM. Few previous studies have used rESWT as a treatment for PDM and even fewer have investigated the brain function of PDM patients from the perspective of imaging.

This study aims to explore the differences in pain intensity, anxiety, depression, mood and functional brain networks in patients with PDM after rESWT treatment. To accurately elucidate the effect of rESWT, we will compare the changes in MRI indexes in the rESWT group and a sham rESWT group and test whether the changes in pain condition are related to changes in brain function after rESWT treatment. Although previous studies have examined brain function in patients with nervous disorders and abnormal change due to PDM, they only explored the disease itself and did not examine the intervention [23, 25, 26]. To our knowledge, our research will be the first to use MRI to reflect divergence of efficacy of rESWT treatment for PDM and the related neural mechanisms through a randomised controlled trial. Existing research [26] shows that midbrain activity is closely related to the Cox retrospective symptom scale (RSS; *r* = 0.489), and the medial prefrontal cortex activity and RSS showed negative correlation (*r* = −0.580); the medial prefrontal cortex aims to reduce the activity of the brain’s pain-regulating system. Thus, the CMSS was chosen as one of the main outcome measures for this study. This scale is usually employed to evaluate dysmenorrhoea symptoms and to detect the severity of dysmenorrhoea.

The limitations of this trial should be mentioned. A sham radial extracorporeal shock wave group will function as the control group in this study. Since no research is available on reliable treatment using sham shock waves, the principle of controlling with no energy output in previous clinical studies is applied. However, limitations occur with any type of sham shock wave. First, this is not a double-blind trial because those conducting the treatment cannot blindly assign treatment without knowing the type of treatment. To minimise the risk of bias, participants and evaluators will be carefully identified for their shock-wave types and trained in advance to apply a sham shock wave with the machine vibrating and pushing onto the handle with almost no energy. Second, a shock wave often shows a large nonspecific effect and produces physiological activity, which cannot be ignored during the experiment. Nonetheless, the neural mechanisms between different groups can be compared. Therefore, by using the MRI results, we can recognise rESWT-specific pathways related to pain amelioration, even if their effects are not significantly different. Third, a healthy group should not be used as a control group. Previous literature has shown differences in the brain networks of patients with PDM and healthy participants [27, 28]. Conversely, our study will focus on the functional brain changes that will be confirmed by extracorporeal shock wave rather than the PDM-specific features against healthy controls. Therefore, the comparison of the changes in brain networks between the rESWT and sham rESWT groups will be emphasised. Thus far, the randomised controlled trial has only been exploratory, but the prospects look promising. In the future, we expect to collect extensive samples with higher-quality randomised controlled trials.

### Trial status

The study was registered with the Chinese Clinical Trial Register (ChiCTR1900020678) on 13 January 2019. Participant recruited was started on 31 January 2019. At the time of submission, seven patients have been selected into our centre. Recruitment is expected to be completed around December 2019.

## Supplementary information

**Additional file 1.** The Standard Protocol Items: Recommendations for Interventional Trials 2013 Checklist.

## Data Availability

Not applicable.

## Abbreviations

AE: Adverse event
ALFF: Amplitude of low-frequency fluctuation
BOLD: Blood oxygenation level-dependent
CMSS: Cox Menstrual Symptom Scale
DPARSF: Data processing assistant for resting state functional magnetic resonance imaging
fMRI: Functional magnetic resonance imaging
PDM: Primary dysmenorrhoea
rESWT: Radial extracorporeal shock wave therapy
SAE: Serious adverse event
SAS: Self-rating Anxiety Scale
SDS: Self-rating Depression Scale
SF-MPQ: Short form of the McGill Pain Questionnaire
